# A QSAR model of Olanzapine derivatives as potential inhibitors for 5-HT2A Receptor

**DOI:** 10.6026/97320630013339

**Published:** 2017-10-31

**Authors:** Pooja Mitra, Aishwarya Rastogi, Mayank Rajpoot, Ajay Kumar, Vivek Srivastava

**Affiliations:** 1 Department of Biotechnology, Rama University Uttar Pradesh, Kanpur, India

**Keywords:** Schizophrenia, 5-HT2A, Receptor, Olanzapine derivatives, AutoDock 4.2, NAMD

## Abstract

Schizophrenia is a complex, chronic mental disorder, affecting about 21 million people worldwide. It is characterized by symptoms, including distortions in thinking, perception, emotions, disorganized speech, sense of self and behavior. Recently, a numbers of marketed drugs for Schizophrenia are available against dopamine D2 and serotonin 5-HT2A receptors. Here, we docked Olanzapine derivatives (collected from literature) with 5-HT2A Receptor using the program AutoDock 4.2. The docked protein inhibitor complex structure was optimized using molecular dynamics simulation for 5ps with the CHARMM-22 force field using NAMD (NAnoscale Molecular Dynamics program) incorporated in visual molecular dynamics (VMD 1.9.2) and then evaluating the stability of complex structure by calculating RMSD values. NAMD is a parallel, object-oriented molecular dynamics code designed for high-performance simulation of large biomolecular systems. A quantitative structure activity relationship (QSAR) model was built using energy-based descriptors as independent variable and pKi value as dependent variable of eleven known Olanzapine derivatives with 5-HT2A Receptor, yielding correlation coefficient r2 of 0.63861. The predictive performance of QSAR model was assessed using different crossvalidation procedures. Our results suggest that a ligand-receptor binding interaction for 5-HT2A receptor using a QSAR model is promising approach to design more potent 5-HT2A receptor inhibitors prior to their synthesis.

## Background

In today world, most of the people are suffering from mental disorder due to many reasons like environmental factors, genetic factors [1] and misbalancing in chemical transmission. Mental disorders have become highly prevalent due to ambitious lifestyle, urbanization, and stressful environment [2]. Mental disorders include major depression, schizophrenia, bipolar disorder, obsessive-compulsive disorder, Alzheimer's disease, anxiety, etc. These disorders can develop at any age and in individuals of any race, religion or income group. Mental and behavioral problems are increasing part of the health problems in all over world. Most of the people are not aware that they are suffering from the symptoms of mental disorder because in the initial stage the symptoms is mild and later on it become a serious mental illness which is very harmful for the society. Schizophrenia is mental health disorder characterized by an array of symptoms, including delusions (fixed false beliefs or suspicions that are firmly held even when there is evidence to the contrary), hallucinations (hearing, seeing or feeling things that are not there), impaired cognitive ability and disorganized speech or behavior [3], affecting more than 21 million people worldwide [4]. Schizophrenia is described in terms of positive and negative symptoms. Positive symptoms are including the delusions disordered, thoughts, speech and tactile, auditory visual olfactory and gustatory hallucinations. The negative symptoms are deficits of normal emotional responses. Schizophrenia etiology indicates that many factors are involved, namely genetic factors, [5, 6]alterations in chemical transmission (dopamine, serotonin etc.,) [7], Obstetrical complications [8] and Viral infections [9]. There is no satisfactory remedy available for prevention of the schizophrenia. Currently available marketed drugs like chlorpromazine, haloperidol, clozapine, risperidone, and olanzapine have nanomolar affinities for dopamine D2 and serotonin 5-HT2A receptors [10] but have some serious adverse effects such as dizziness, diabetes, weight gain, neuroleptic malignant syndrome, sexual dysfunction, agitation and sedation. To treat positive as well as negative symptoms of Schizophrenia atypical antipsychotics drugs focused more on 5-HT2A receptor instead of D2 dopamine to avoid side effects called extrapyramidal symptoms (EPS). Neurotransmitter serotonin (5- hydroxytryptamine; 5-HT) an ancient neurotransmitter, involved in several neurophysiological and behavioral functions, acts by interacting with multiple receptors (5-HT1-5-HT7) [11]. Its functions are expressed in cardiovascular, gastrointestinal and central nervous systems. Alterations in serotonergic signalling have also been implicated in various psychiatric disorders [11]. Drug resistance in schizophrenic disorders treated with an antipsychotic medication is highly problematic, lacking sound criteria to define it, and to discriminate between drug response and clinical remission. Several neurochemical abnormalities have been reported to be relevant for the pathogenesis of schizophrenic disorders and have been related to clinical symptoms as well as to the quality of response to antipsychotics: most of the findings come from studies on, dopamine D2 and serotonin 5-HT2A receptors, brain metabolism, but more recently, other non-dopaminergic pathways have been implicated. Nowadays, molecular docking approaches are routinely used in modern drug design to help understand drug receptor interaction. It has been shown in the literature that these computational techniques can strongly support and help the design of novel, more potent inhibitors by revealing the mechanism of drug receptor interaction. However, so far, there has been no report concerning the application of molecular docking methodology for understanding the binding of Olanzapine derivatives [12]. In this study, we docked experimentally verified 11 Olanzapine -based inhibitors having inhibitory value pKi with 5-HT2A receptor using AutoDock 4.2, which resulted in energy-based descriptors. Molecular dynamics (MD) simulation studies of inhibitor - protein complex were performed by NAMD. Recently, more advanced techniques have attempted to model the receptor environment for accommodating ligand structure. QSAR studies incorporate data for ligands and provide a more detailed analysis of ligand receptor interactions [13]. We have build quantitative structure activity relationship (QSAR) model using multiple linear regression analysis.

## Methodology

### Protein target structure:

The 3D coordinates of the crystal structure of the LSD-bound 5- HT2B retrieved from Protein Databank (http://www.rcsb.org/). This is used docking. The structure was optimized using the chimera tool [14].

### Model building and Evaluation

The amino acid sequence of 5-hydroxytryptamine receptor 2A (Entry No.: P28223) was retrieved from UniProtKB database (http://www.uniprot.org/) and taken as target protein sequence. The modeling of 3D structure of target protein followed a stepwise procedure, starting with a template structure search from PDB (http://www.rcsb.org/pdb/), related to the target sequence using BLASTP [15]. From a number of hits, a potential template structure (PDB-ID: 5TVN), representing the Crystal structure of the LSD-bound 5-HT2B receptor was taken as template for model building. The template and target sequence was aligned using the align2d script available in MODELLER 9v18 [16]. Based on the alignment, five comparative models of the target sequence were built by MODELLER. The best model can be selected by picking the model with the lowest value of the Modeller objective function and DOPE (Discrete Optimized Protein Energy) score from a collection of models built by MODELLER. PROCHECK [17] check the stereo-chemical qualities of the model.

### Inhibitors dataset

Eleven Olanzapine derivatives with known pKi were obtained from literature [18]. Derivatives build using PubChem Sketcher V2.4 [19] and save in smiles format. The 3D structures of known 11 inhibitors were building using CORINA V3.6. Molecular Networks GmbH Computerchemie maintains CORINA for general usage. All the ligands were subjected to energy minimization using the HyperChem software [20].

### Molecular docking:

Molecular docking is a key tool in structural molecular biology and computer-assisted drug design. The goal of ligand-protein docking is to predict the predominant binding model(s) of a ligand with a protein of known three-dimensional structure [21]. Docking of eleven olanzapine derivatives screened from literature against 5-HT2A Receptor structure were done using molecular docking program AutoDock [22]. Gasteiger charges are added to the ligand and maximum 6 numbers of active torsions are given to the lead compounds using AutoDock tool [23]. Kollman charges and the solvation term were added to the protein structure. The Lamarckian genetic algorithm implemented in Autodock was used for docking.

### Molecular dynamics simulations

Molecular dynamics simulations were done using the NAMD [24] incorporated in visual molecular dynamics (VMD 1.9.2) [25]. The protein-ligand complex was immersed in the center of a 50 Å box of water molecules where all water molecule atoms (H-O-H) were closer than 1.5 Å and a CHARMM22 parameter file for proteins and lipids; phi and psi cross-term map correction were used in the force field for complexes. A protein structure file (psf) was created from the initial pdb and topology files using psfgen package of VMD. After running psfgen,two new files were generated protein pdb and protein psf and by accessing PSF and PDB files; NAMD generated the trajectory DCD file. After the simulations, the results were analyzed in VMD by calculating the Root mean square deviation (RMSD) of the complex using rmsd tcl source file from the Tk console and finally rmsd.dat was saved and accessed in Microsoft office excel 2007.

### 2D QSAR

A QSAR based model was generated having correlation coefficient r2 value 0.63861 was developed using multiple linear regression analysis. An equation was developed for the inhibitory activities represented as pKi values using the six types of energy values as variable descriptors such as Binding Energy (BE), Intermolecular Energy (IME), Internal Energy (IE), Torsional Energy (TorE), vdW + Hbond + desolv Energy (VdwE) and electrostatic energy (EE). A correlation coefficient (r2) of 0.6386 was obtained for 11 olanzapine derivatives as shown below in equation 1.

Predicted pKi = 26.37776 - 27.89117089 (BE) + 157.8919 (IME) - 5.85273 (IE) +25.42601 (TorE) - 128.136 (VdwE) -130.266(EE) (1)

Several cross-validation procedures were adopted to assess the predictive performance of the QSAR model. In leave-one-out strategy (LOOCV), one molecule was removed from the dataset as a test compound and the remaining 10 molecules were used to build the model. This process was repeated 11 times with each inhibitor as a test molecule.

## Result and Discussion

**Figure 1:**
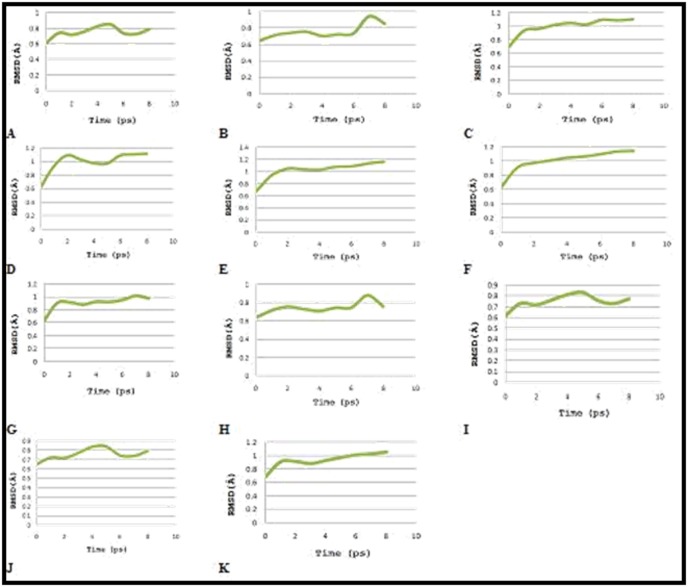
Graph displaying root mean square deviation (RMSD) of (A) derivative 1 (B) derivative 2 (C) derivative 3 (D) derivative 4 (E) derivative 5 (F) derivative 6 (G) derivative 7 (H) derivative 8 (I) derivative 9 (J) derivative 10 (K) derivative 11 - 5-HT2A receptor complex versus time (5 ps) at 310 K, resulted in highest peak at 0.85, 0.94, 1.10, 1.12, 1.16, 1.14, 1.02, 0.88, 0.83, 0.84 and 1.06 Å respectively.

Modeller 9.18 generated the 3-D structure of the 5-HT2A receptor with the help of template model 5TVN. The best model has Modeller objective function 895.70007 and -22964.48242 as DOPE score. Homology model of 5-HT2A receptor was validated with Ramachandran plot analysis through PROCHECK, and observed that 91.4% of the residues were in most favored regions. Based on R1, R2, R3 and R4 groups at different positions, olanzapine derivatives of 5-HT2A Receptor were retrieved from literature [18] and are shown in Table 1. In docking studies of olanzapine derivatives with 5-HT2A Receptor, best autodock score was used as criteria to interpret the best conformation among the 30 conformations, generated by AutoDock 4.2 program. The docking result of the olanzapine derivatives with 5-HT2A receptor was shown in Table 1. Further, the docked complexes were analyzed through Python Molecular Viewer software [26] for their interaction studies. Thus from the complex scoring and binding ability it's deciphered that these compounds are promising inhibitors for 5-HT2A receptor. Therefore, the constructed 3D model of protein-ligand complexes was processed for MD simulation for a 5ps timescale with Langevin dynamics to control the kinetic energy, temperature, and/or pressure of the system. The RMSD values of complexes contain alpha carbon atoms, and all atoms were calculated by taking structure with reference conformation points. The RMSD values of complex versus time were shown in Figure 1. Relationship between experimental and predicted pKi values of Olanzapine derivatives was shown in Table 2.

## Conclusion

A QSAR model using pKi values for eleven known olanzapine derivatives binding with 5-HT2A receptor as dependent variable and molecular docking based predicted pKi with a correlation coefficient r2 is 0.63861 was reported. The 2D-QSAR results revealed some important information as discussed in result. This study may be identify new compounds through virtual screening and predict the bioactivity of new compounds. The quantitative structure-activity relationship (QSAR) and molecular modeling studies have been increasingly employed in rational drug discovery process to understand the drug receptor interaction and to design new molecules with higher potency [27]. Thus useful clues to designing novel inhibitors of 5-HT2A receptor with high affinity for the treatment of Schizophrenia.

## Conflict of interest

The authors declare that they have no conflict of interest.

## Figures and Tables

**Table 1:** Olanzapine derivatives of 5-HT2A receptor on the basis of different R1, R2, R3 and R4 group.

Olanzapine derivate	Group			
R1	R2	R3	R4
1	CH3-CH2	H	CH3	H
2	CH3	CH3	CH3	H
3	CH3	C3H7	CH3	H
4	CH3	isobutyl	CH3	H
5	CH3	H	CH3	CH3
6	C6H5	H	CH3	H
7	CH3	H	H3C-CH2	H
8	CH3	H	C3H7	H
9	CH3	H	CH2F	H
10	CH3	H	CH2Cl	H
11	CH3	H	CH2OH	H

**Table 2:** Docking results of Olanzapine derivatives with 5-HT2A receptor structure with activity (pKi = - logpKi)

S.No	Experimental pKi	Predicted pKi	BE	IME	IE	TorE	VdwE	EE
1	8.15	8.46	-10.11	-10.41	-0.13	0.3	-9.72	-0.69
2	8.27	8.33	-10.02	-10.32	-0.09	0.3	-9.66	-0.66
3	9.29	9.32	-9.77	-10.36	-0.38	0.6	-9.78	-0.58
4	9.19	8.46	-10.28	-10.87	-0.36	0.6	-10.19	-0.68
5	8.69	8.28	-9.33	-10.23	-0.48	0.89	-9.93	-0.3
6	7.85	8.18	-11.69	-12.29	-0.83	0.6	-11.66	-0.63
7	7.61	8.21	-9.09	-10.28	-0.52	1.19	-9.86	-0.42
8	7.51	5.86	-9.18	-10.67	-0.61	1.49	-10.31	-0.36
9	7.71	7.71	-9.3	-10.2	-0.52	0.89	-9.69	-0.5
10	8.61	8.32	-9.31	-10.21	-0.58	0.89	-10.18	-0.03
11	7.21	7.49	-8.73	-9.92	-0.44	1.19	-9.84	-0.08

BE = Binding Energy; IME: Intermolecular Energy; IE = Internal Energy; TorE= Torsional Energy; VdwE = vdW + Hbond + desolv Energy; EE= Electrostatic energy.
